# Staff Expectations of an Australian Integrated Model of Residential Rehabilitation for People With Severe and Persisting Mental Illness: A Pragmatic Grounded Theory Analysis

**DOI:** 10.3389/fpsyt.2019.00468

**Published:** 2019-07-08

**Authors:** Carla Meurk, Stephen Parker, Ellie Newman, Frances Dark

**Affiliations:** ^1^Policy and Epidemiology Group, Queensland Centre for Mental Health Research, Wacol, QLD, Australia; ^2^School of Public Health, The University of Queensland, Herston, QLD, Australia; ^3^Rehabilitation ACU, Metro South Addiction and Mental Health Service (MSAMHS), Brisbane, QLD, Australia

**Keywords:** community care unit, implementation, qualitative methods, rehabilitation, peer support, schizophrenia

## Abstract

Mental health services increasingly involve peer support workers. Staff expectations of working in these services are important because they frame processes and cultures that develop within services, and influence work satisfaction, staff retention, and consumer experience. We examined staff expectations at two new community-based residential rehabilitation units trialing a staffing model where most staff are employed based on their lived experience of mental illness. Qualitative semi-structured interviews were conducted with ten peer support workers and five clinical staff on commencement at Community Care Units that opened in 2014 and 2015. Staff views covered individual motivations, emerging organizational practices and culture, and the nature and philosophy of recovery and recovery-oriented rehabilitation. Subtle differences were evident in staff understandings of recovery and recovery-oriented rehabilitation. Staff were mostly optimistic about the services’ potential but expressed uncertainty about how the professions would work together and practicalities of the new roles. Concerns that staff foreshadowed are consistent with those reported in the literature and can be pre-emptively addressed. Future research on staff experiences will enhance understanding of how staff perceptions of recovery-oriented rehabilitation change over time, and of how these relate to consumer experiences and outcomes.

## Introduction

Community care units (CCUs) intend to deliver community-based recovery-oriented transitional residential mental health rehabilitation, predominantly to those with a severe and persistent mental illness (1, 2). Literature exploring staff experiences of working at these units has suggested ongoing tensions and uncertainty between recovery concepts and rehabilitation practice (3, 4). One way that rehabilitation services have attempted to realize recovery-oriented practice is by incorporating peer support roles into their model of service (2, 5, 6).

### Recovery-Oriented Practice

Recovery and recovery-oriented practice are central tenets of mental health policy and services delivery in Australia (7). Recovery concepts have numerous interpretations and can be challenging to implement (8). One definition of recovery is as “a deeply personal, unique process of changing one’s attitudes, values, feelings, goals, skills, and/or roles (and) a way of living a satisfying, hopeful, and contributing life even with limitations caused by illness” (9, p. 527 10, p. 12). Kidd et al. (11) have identified the importance of partnerships in delivering recovery-oriented care, while Jacob et al. (12) highlight the value of “self-focused” care for consumers with lived experience of mental illness.

### Integrating Peer Support Roles Into Recovery-Oriented Rehabilitation

There has been widespread, albeit incomplete (13), support for the value of integrating peer support roles into mental health service delivery to enhance recovery-oriented practice (2, 10, 14). Developing the peer support workforce and incorporating peer-based interventions into routine care is endorsed in Australian mental health policy (7). A study exploring consumers’ expectations of a CCU found favorable expectations of an integrated staffing model with regards to peer workers being “people you can relate to,” facilitating the “breaking down (of) traditional barriers,” and fostering a more positive and hopeful environment (15, p. 1,656). Yet, establishing a sustainable and meaningfully integrated peer support workforce in routine service delivery is challenging (14) and diverse approaches to involving peers have emerged (2, 5, 10). Understandings of these challenges is limited but improving (**Table 1**).

**Table 1:** Facilitators and barriers to implementing peer support roles within mental health services.

Facilitator/barrier	Locus	Implementation impact	Reference
Vulnerabilities and/or care needs of peer support workers	Individual	Undermines	(14, 16, 17)
Professionalism of peer support workforce, including credentialing	Individual	Contested	(14, 17–21)
Role clarity and distinctiveness (including provision of training)	Institutional	Supports	(14, 19–22)
Strategic alignment of peer support with service goals	Institutional	Supports	(22)
Valuing the contribution of peer roles; recognizing their credibility	Individual	Supports	(14, 17, 20, 21)
Discrimination against peer support workers	Individual	Undermines	(23)
Training of non-peer staff (including anti-discrimination training)	Institutional	Supports	(14)
Sufficient numbers of peer roles	Institutional	Supports	(14)
Systematic approach to implementation of new roles, and appropriate resourcing	Institutional	Supports	(20, 21)
Shared expectations	Relational	Supports	(22)
Boundary issues, including dual (personal-professional) relationships	Relational	Undermines	(18, 19)
Role conflict between consumer and non-consumer providers	Relational	Undermines	(18, 19)
Strong adherence to medical model	Individual/Institutional	Undermines	(24)

The aim of the present paper was to analyze qualitative interviews undertaken with staff at two new CCUs trialing a staffing model incorporating peer support about their understandings and expectations of working in recovery-oriented rehabilitation services in an Australian setting.

## Methods

This paper comprises one component of a longitudinal mixed methods comparative evaluation of the equivalence of an integrated peer-support and clinical staffing model for residential mental health rehabilitation; specifically, this paper presents the qualitative analysis of staff understandings and expectations of working in a recovery-oriented rehabilitation service that was trialing an integrated peer-support model, at commencement. The published protocol provides comprehensive methodological detail (25) and reporting of the study’s methods and findings follow the Consolidated Criteria for Reporting Qualitative Research (COREQ) guidelines, as applicable (26).

### Study Sites

CCUs support people with severe and persisting mental illness to achieve personal recovery goals over a 6–24 month timeframe. Most consumers will have a diagnosis of schizophrenia or a related psychotic disorder (27). This study was undertaken at two new outer-suburban CCUs located within a large public mental health service in Brisbane, Australia. These CCUs were an addition to the existing mental health service array that included acute and sub-acute inpatient care; step-up/down community residential care; community case-management, outpatient drug, and alcohol services and rehabilitation teams.

The CCUs began operation in December 2014 and January 2015 and are trialing a novel integrated staffing model where most staff are employed as peer support workers (PSWs) based on their lived experience of mental illness (2). The aim of this novel staffing model was to combine lived experience and therapeutic lenses to facilitate the collaborative development of effective rehabilitation plans with consumers. The staffing model was not intended to alter the core rehabilitation function of the CCUs. Staffing profile and site characteristics are summarized in **Table 2**.

**Table 2:** Characteristics of study sites.

		Site 1	Site 2
*Staffing*	Total FTE staff	24.5	18.4
	Total FTE peer-support staff	16	10.4
	Total FTE clinical staff	7.5	7
	Peer support: clinical staff ratio	2.13	1.49
	Staff: consumer ratio	1.2	1.2
*Physical environment*	Maximum occupancy (consumers)	20	16
	Number of self-contained independent living units	20	14
	Number of dual-occupancy independent living units	0	1
*Philosophy of care*	Recovery-oriented	Yes	
	Strengths-based	Yes	
	Designated rehabilitation focus	Yes	
	Voluntary engagement in rehabilitation^	Yes	
	Individualized care planning	Yes	
	Transitional support	Yes	
*Available treatment and support*	Individual psychotherapy support cognitive behavior therapy	Yes	
	Living skills support and development	Yes	
	Structured leisure and physical activities	Yes	
	Evidence-based therapeutic group programs	Yes	

^Consumers subject to involuntary treatment orders are accepted on the basis of voluntary consent and participation

At a CCU consumers reside in self-contained, independent living units in an apartment complex with 24-hour support provided by a multidisciplinary team who assist them with living skills development and community re-integration. Available therapeutic programs at the study sites include cognitive behavior therapy, cognitive remediation, and social cognitive interventions. The philosophy of care documented in the model of service for the CCUs acknowledges the possibility of recovery and aims to provide recovery-oriented and rehabilitation focused care (2). Residential support is transitional and strengths-based, aiming to facilitate self-determination through individualized care planning and voluntary engagement in rehabilitation activities of relevance to consumers’ goals (2).

### Sample and Data Collection

Ethical clearance was granted by the Metro South Human Research Ethics Committee (HREC/14/QPAH/62). All staff were approached at the commencement of operation at the respective site to provide voluntary informed consent to participate. Convenience sampling was used to allocate the order of participation, with interviews being prioritized based on the order in which consent was provided and availability at interview times. Sampling continued until it was deemed that thematic saturation was reached. Interviews were completed between December 2014 and March 2015; all occurred within the first 6 weeks of commencement of operation at the respective site. Semi-structured interviews were completed by an independent interviewer (EN). Interviewer independence aimed to support an open and candid discussion.

The interview schedule explored three topics: how staff thought the experience would compare to previous mental health settings where they had worked; expectations of the CCU; and why they had chosen to work there. To avoid leading participants to discuss recovery concepts, the interviewer was instructed not to use the term “recovery” unless it was introduced by the interviewee. If participants used the term “recovery,” the interviewer followed up with a prompt to ascertain their meaning of the term. In this way, the pertinence of the concept and how its meaning may vary among staff was explored. Interviews were audio-recorded and transcribed verbatim. Transcripts were returned to staff for review and approval. De-identified transcripts were uploaded to NVivo11 for analysis (28).

### Analysis

A pragmatic grounded theory approach was taken, described in full elsewhere (25, 29). Data collection, analysis, and theorizing occurred concurrently (30). After three interviews at each site, the research team considered the emerging themes, coding framework, adequacy of the interview schedule, and estimated the sample size likely to achieve thematic saturation.

SP developed and applied an initial coding framework to the data, which CM refined and revised. The team then explored limitations in the coding and the theory’s grounding in the data. A comprehensive appraisal of prominent and subordinate content and themes was undertaken, with a view to facilitating future comparison of staff expectations and experiences in the setting. Attention was paid to exploring systematic differences—between sites and between clinical and PSW roles. Before finalizing results, all current CCU staff were invited to listen to, and give feedback on, a presentation of preliminary findings. Twenty-two current CCU staff (9 PSWs and 13 clinical staff) elected to attend the feedback session, which provided a means of validating and refining findings, and for group reflection on the implications of these. No major discrepancies arose between authors’ and staff interpretations, and staff feedback was incorporated into the final analysis.

## Results

### Sample Characteristics

Thematic saturation was achieved following interviews with 15 staff members. Ten were PSWs and five were clinical staff (nursing, social work, and occupational therapy). The sample comprised approximately one-third of the staff commencing (N = 46) and was broadly representative of the staffing profile, where PSWs comprise 64% of full-time equivalent roles (25). Ten interviewees were female, two interviewees had previous experience working within a CCU (one PSW and one clinical staff), five PSWs, and three clinical staff had previous experience delivering mental health support in the non-government sector. The mean interview duration was 34 min (median = 36, SD = 6.6 minutes). Two staff elected to edit their transcripts prior to analysis, both redacting and providing additional information. No major content or thematic differences were identified, either across sites or staffing roles, so data were analyzed together.

### Conceptual Model

**Figure 1:**
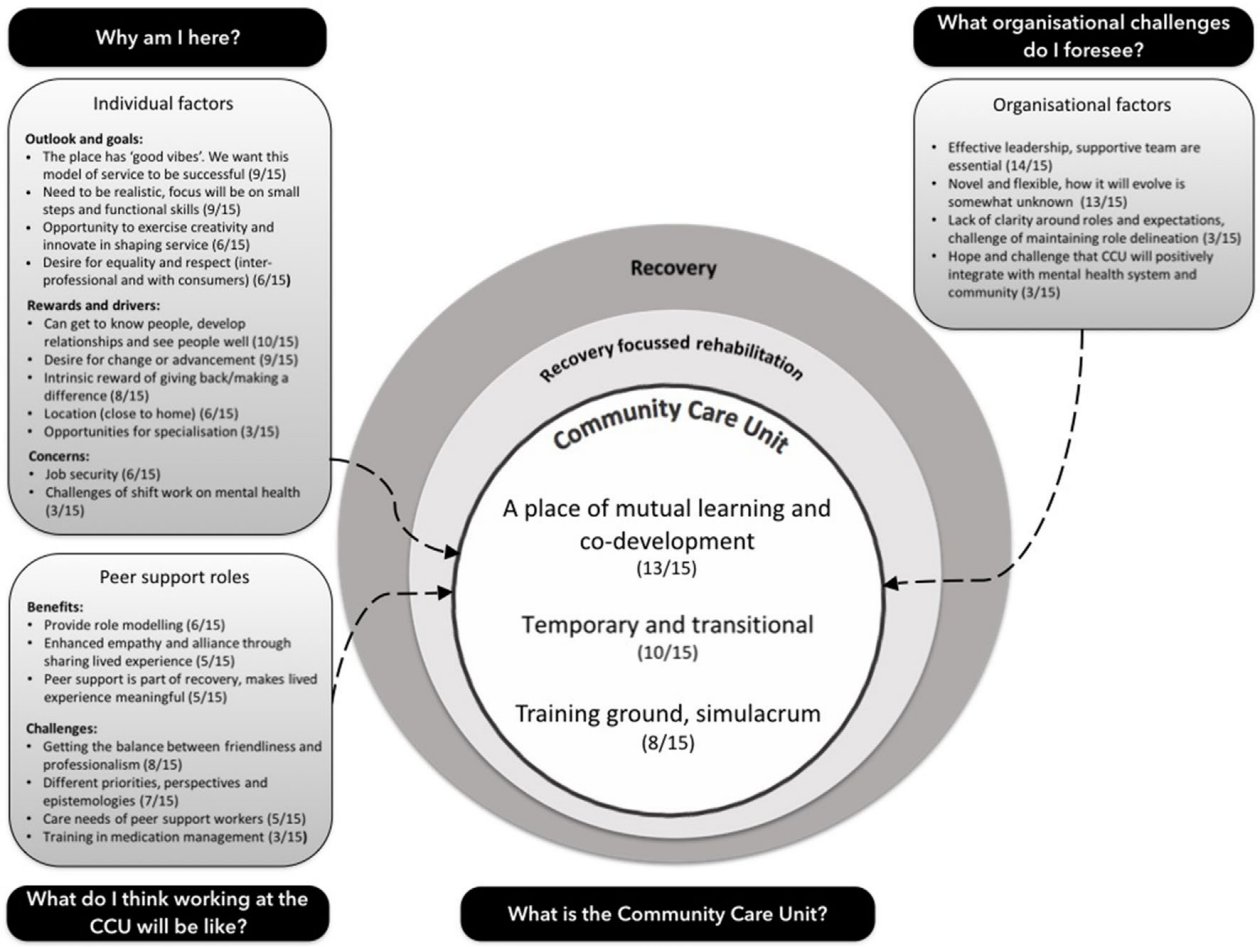
Conceptual model arising from qualitative interviews of staff expectations.

**Figure 1** visually depicts the conceptual model that encapsulates the study’s findings. Topics, themes, and sub-themes within this model are described, elaborated, and analyzed, with representative extracts, in the subsequent sections.

### Perspectives on the Model of Service

#### What Recovery Means

The term “recovery” was mentioned in 12 out of 15 transcripts. Six staff provided an explicit or in-depth discussion of the concept (**Table 3**). Other participants referenced related concepts as part of the broader discussion, and process based and individualistic accounts of recovery predominated. Staff expressed the idea that recovery is a journey (“journey” appeared in nine transcripts). Respect for individuality and the importance of person-centered care were also prominent, appearing in 10 transcripts. Conversely, the concept of “meaning” (for example, the importance of building a meaningful life) was infrequently discussed, with the word “meaningful” itself being used in the context of recovery in four cases. Similarly, clinical concepts of recovery were infrequently referenced; two clinical staff described recovery in the context of managing mental illness or its symptoms. Only three peer staff mentioned the term “symptoms”: one in the context of their own recovery; one in the context of describing the management of symptoms as one kind of recovery; and one who described such a focus as an old idea that had been superseded by holistic and non-medicalized notions of recovery. Notions of recovery as a perpetual and transformational cycle were alluded to by two staff, and one staff member alluded to recovery involving re-integration within the community.

**Table 3:** Transcript extracts relating to staff concepts of “recovery”.

Recovery is… “A journey” [INTSTA076-PEER] “A personal journey” [INTSTA061-PEER] “Individualized to the person” [INTSTA004-CLIN] “About living a life that’s meaningful to you” [INTSTA076-PEER] “Where they want to be at not to where I think they, you know, should be at.” [INTSTA081-PEER] “Learning how to better manage, with their symptoms and then in everyday life as well” [INTSTA053-CLIN] “Actively [living] with [a] mental health problem in a way that [ … ] enhances [ … ] quality of life.” [INTSTA045-CLIN]

#### What Recovery-Oriented Rehabilitation Means

**Table 4** summarizes sub-themes regarding what recovery-oriented rehabilitation means. The predominant sub-theme in accounts of recovery-oriented rehabilitation was that of empowering residents to control their own recovery and develop self-reliance (9/15). Consistent with staff understandings of recovery, recovery-oriented rehabilitation was viewed as enabling residents to make choices and set their own goals. This was linked to the notion that the CCU provided a ‘safe-space’ and created opportunities for residents to take risks and make mistakes:

[P]eople need to be able to make mistakes and [ … ] make that choice [ … ] then when they do make the appropriate choices for themselves they’re the ones that have made them and they’re the ones that have owned it. [INTSTA050-PEER]

**Table 4:** Staff concepts of “recovery-oriented rehabilitation”.

Recovery-oriented rehabilitation means…	Representative transcript extracts
Empowering residents in controlling their recovery and developing self-reliance (9/15)	[W]e’d go to them and go, ‘so what do you want to do today?’, and they’d go, ‘well what do you mean?’ I’d go ‘well, what’s your plans?’ not like, ‘I’ve got plans for you’; ‘you’re going to tell me what you want to do’. [INTSTA079-PEER] I really hope that we can look at ways that we can help people do—do things for themselves, you know. [INTSTA076-PEER] [I]t’s giving them the, the skills, the resources, the, you know, whatever, you know, coping mechanisms, whatever it is to, you know [ … ]. They’ve learnt those skills and, um, strategies to do it on their own eventually. [INTSTA081-PEER]
Focusing on small steps and functional skills (mastering the challenges of everyday living) (6/15)	[J]ust breaking things down a lot more into smaller, gradual steps over a longer kind of period I would imagine. [INTSTA045-CLIN] [Working] on like personal skills and getting them more comfortable with dealing with other people and getting them out into the community. [ … ] also a lot of functional [ … ] processes [ … ] your basic budgeting and cooking and things like that to get them to where they need to be um independently. [INTSTA048-PEER]
Participating in residents’ recovery journey (6/15)	I’m looking forward to walking beside the residents and [ … ] the community participation. [ … ] I don’t want to ever tell them what to do, or be bossy with them—and—and what’s going to be the balance between trying to motivate them—and—and still be their peer. [INTSTA016-PEER] I’m really excited to, sort of, become part of their journey in recovery. [INTSTA061-PEER]
Having a strengths and self-esteem focus (4/15)	I think it’s working with the consumer where they’re at at the present moment and trying to find the strengths that they have inside of them and their abilities to maybe build on that. [ … ] so they have skills and access to additional resources for a future that provides them with a greater sense of self-worth and engagement with their community. [INTSTA045-CLIN]

Some staff expected that a key part of their roles would be helping residents develop life skills (6/15), including interpersonal skills and living skills like budgeting and cooking. One staff member described that they thought it was important that life at the CCU emulated and provided training for the realities that residents would face when they left:

What are we going to do, we’re going to go to the shops, okay. How are we going to get there? We’re not using the car. Because that’s the first thing they think, let’s get in the car. You think no that’s not going to happen in the real world. So, okay, so then just finding out what, what bus—up in town there’re so many buses going every different way. [INTSTA079-PEER]

#### What a CCU is

Staff understood that the CCU is a service designed to realize a recovery philosophy and deliver recovery-oriented rehabilitation. Three sub-themes underpinned this conception, with the CCU being considered: a place of mutual learning and co-development (13/15); a temporary and transitional place (10/15); and a training ground and simulacrum (or “model reality”) of community living (8/15).

1. CCU as a place of mutual learning and co-development
  The sub-theme of learning was prominent in nearly all staff accounts (13/15). Staff described how they hoped to learn from other staff and residents, and for other staff and residents to learn from them and each other:
  [W]hat I’m hoping for and I suppose that’s what I’m working from my individual practice from at the moment is um is that openness to receive constructive criticism and receive kind of direction um and also to be able to reciprocate that with others too. [INTSTA045-CLIN]
  For a small number of participants, the ethos of the CCU as a place of learning extended to its role as a place of research and development (2/15), with one staff member identifying that they were ‘excited’ about the research taking place within the CCU [INTSTA032-CLIN].
2. A temporary and transitional place
  Most staff (10/15) identified that the CCU provided a temporary and transitional place—in their own words “a pit stop or a check in point” [INTSTA074-PEER]. Staff articulated the idea that the CCU was a place and point in time that could be transformative for residents, including those who were transitioning from acute and long-term care settings to the community. Consistent with the sub-theme of CCU as a place of learning, one staff member stated that they would know they had made a difference when they saw “residents graduate” [INTSTA022-PEER]. Another staff member highlighted the importance of not fostering dependency [INTSTA076-PEER].
3. A training ground and simulacrum (i.e., model reality)
  Elaborating on the idea of the CCU as “a training ground” [INTSTA050-PEER] for community living, over half (8/15) of staff emphasized that the CCU should seek to be a model of the “real world.” One staff member described the CCU as:
  [A] community-based setting and environment. It’s kind [of] a home-like environment [ … ] I think it helps to build that more human state kind of element to it. Um yeah and makes it a bit more personable rather than kind of clinical. [INTSTA045-CLIN]
  This idea—and the positive value attributed to it—that the CCU provides a “natural” setting rather than a contrived hospital or clinical setting, was linked with the idea that it would be easier for staff, particularly PSWs, to build a “natural rapport” [INTSTA004-CLIN] with residents.
  I think that for the residents, it should feel much more natural, whereas rather than feeling that they’re constantly being assessed all the time by clinicians, that the peer support are actually doing that, and that the clinicians are listening to the peer support, to pick up those cues of clinical aspects. [INTSTA004-CLIN]
  One PSW endorsed the hope that the CCU would function as “this really awesome learning environment that’s not—that’s really like always really organic [ … ]” [INTSTA016-PEER]. Yet, they also expressed uncertainty as to whether their role would come naturally:
  I’m not exactly sure yet if it’s gonna come about in a really natural way or if I’m—I’m gonna have some strategy in my approach. [ … ] and I think um it might be a combination of two that develops over time. [INTSTA016-PEER]
  The importance of clinical care, including medication and medication management, was not widely considered (3/15). Those who did discuss it were positive about the prospects of integrating social, psychological, and medical care in one setting:
  I think that’s going to—it’s going to work well because, um, it sort of means that people are getting the—the sort of support that they need with the, um, the social, the psychological, and the—the medical [ … ] in the one setting. [INTSTA076-PEER]

### Individual Level Factors

#### Outlook and Goals

Staff were generally optimistic about the CCU, describing it as having “positive energy” and “good vibes,” and expressing how much they wanted the service to be a success (9/15):

I like the vision of this place [ … ] the vibe you get off everyone, like just the mood [ … ] like when I walk in I feel immediately better. [ … ] like it’s just so positive here [ … ] [INTSTA094-PEER]

For most staff (9/15) this optimism was tempered by realism. Staff expected to encounter challenges, including challenges relating to the side effects of psychoactive medications.

I think you have to be realistic and understand the impact that medications have and the demotivation [ … ] like the engagement is still going to be an ongoing issue [INTSTA053-CLIN]

In recognizing that challenges lay ahead, staff indicated that they were prepared to “rejoice” in small successes [INTSTA004-CLIN].

Egalitarianism permeated interviews. Some staff expressed a desire for equality and respect in their roles within the new service, both inter-professionally and with residents (6/15).

I would hate to think that some people might—some residents might get preference because, you know, they’re more engaged and personable, easy to like. Whereas you might get someone that’s a little bit more difficult with their personality and might be reluctant to engage for whatever reason. [INTSTA053-CLIN]

I would hate to see, you know, one side [clinical or peer support … ] out power the other [ … ]. It needs to be completely sort of equal. [INTSTA076-PEER]

Some staff (6/15) identified that working in a new service allowed for flexibility in how the services would evolve. Some indicated that they were looking forward to the opportunity to exercise creativity and innovatively shape the service or identified that they had no expectations and would ‘go with the flow’ [INTSTA081-PEER]. Others were wary about what might transpire, for example, that the CCU risked becoming an accommodation service [INTSTA053-CLIN].

#### Rewards and Drivers

Some staff identified a variety of intrinsic and pragmatic rewards and drivers associated with working at the CCU and hoped to forge different, stronger, and more equitable relationships with residents (6/15). One staff member described that they thought it a “privilege” [INSTA032-CLIN] to participate in a resident’s recovery. Most staff looked forward to the opportunity to get to know residents, to develop relationships and, crucially, to see people well (10/15).

In the inpatient unit, you know, they’re going through really quickly [ … ] they’re acutely unwell and so you’re just trying to get the, through that bad phase [ … ]. And then, you know, the next one comes through. [ … ] [Y]ou didn’t see [ … ] what could be. [INTSTA079-PEER]

Most staff wanted to make a positive difference (8/15), and in one case a PSW described their role as “giving back” to the health system that had helped them.

I felt as if, yeah, I’m in a position with my health where I can help people. [ … ] not only giving back to the community and the consumers, like helping them, sort of giving back to the services, like Queensland Health, from when I went into the public mental health place [INTSTA094-PEER]

Pragmatic drivers included a desire for vocational change or advancement (9/15), the location of the new service (i.e., being closer to home) (6/15), and opportunities the CCU offered for role specialization (3/15).

#### Concerns

Two personal level concerns were voiced by staff: job security (6/15) (PSWs were employed on 24-month fixed term contracts); and the challenges of shift work and its possible negative impacts on mental health (3/15).

### Organizational Factors

Nearly all (13/15) staff spoke about the novelty of the CCU model and its capacity to evolve in unforeseen ways. There was nearly unanimous belief as to the importance of effective leadership and a supportive team environment to the successful functioning of the CCU (14/15). Staff wondered how the multidisciplinary team would come together, recognizing this as a collective responsibility; they hoped a cohesive team would emerge over time.

[I’m] hoping that this team comes together. That there’s not going to be divides. [ … ] whether it’s various disciplines *versus* various disciplines, or clinical *versus* non-clinical, or whatever, I’m wary that there can be divides form from time to time and I’m just—I’m just hopeful that doesn’t happen here. [INTSTA032-CLIN]

Three staff (3/15) discussed a lack of clarity around roles and expectations or that they thought it would be challenging to maintain role delineation overtime.

[ … ] I know in some of these environments, like it can be really hard to distinguish [ … ]. So I think letting everyone have a voice but knowing—everyone knowing what their exact role is ‘cause I think that can be really blurred a little bit. [INTSTA048-PEER]

Three staff (3/15) expressed the hope that the CCU would integrate with, and be viewed positively by, the mental health services sector and the broader community.

I want it [the CCU] to be seen as a positive thing within the community and the mental health community and, um, yeah that’s the goal anyway [INTSTA053-CLIN]

### Peer Support Roles

#### Benefits

Having PSWs within the service was described by some staff as an opportunity for clinical staff and PSWs to role model effective relationships (6/15).

Yeah definitely that you don’t have the, such a power imbalance then and they’re not being given therapy as such from peer workers. It—it’s almost like a model of the yeah just a healthy relationship, everyday relationship. [INTSTA050-PEER]

In particular, some staff viewed that sharing lived experience was a means to facilitate greater empathy and alliance (5/15).

I think the motivation of the staff here, ah—I won’t say better, but I think it would be different, because we can have that lived experience, that people will be able to ah, empathise and sympathise and—and be able to say to our consumer, this is my story and I was here [ … ] and I think that hope that that can give to consumers will be ah, beneficial here [INTSTA004-CLIN]

Some staff identified that PSW roles afforded an opportunity for those with lived experience of mental illness to view this as a strength and a valued tool, rather than a hindrance to employment. Some also saw it as part of their recovery and that it contributed to making their journey meaningful (5/15).

[ … ] like in that moment that I read the job description, um my past sort of made sense and didn’t feel like I’d just been wasting my time. It felt like I’d been doing infield work. [INTSTA016-PEER]

#### Anticipated Challenges Associated With the Integration of Peer Support Roles

Several potential challenges of PSW roles were identified. Chief among these was the expected challenge of achieving a balance between friendliness and professionalism (8/15). Some staff foresaw difficulties if boundaries between “professional” and “familiar” relationships were crossed.

[ … ] some of the peer support there were saying, you know, how much, um, personal information should you really divulge, because they have never worked in this role before. And you know, we were trying to explain [ … ] don’t want to burden your patient with your problems, so whatever you do, you know, there’s a fine line between that, you know. So, don’t have them counselling you—like you’ve got to [be] helping them. [INTSTA079-PEER]

[T]hat we do have policies and procedures, so that they don’t become too friendly with the consumer in a—in a boundary issue way. Ah, so for example, if someone’s smoking in their unit, I want them to tell me, because it is breaking one of the rules. I don’t want them to think that was going to affect their relationship with the consumer. [INTSTA004-CLIN]

As described earlier, the idea of the CCU as a place of learning—including across disciplines and perspectives—was described as a key advantage of the CCU, and something that staff looked forward to. However, successful integration of these perspectives and practices was also described by some as a challenge for the service to manage (7/15), particularly with respect to differences that might exist between peer and non-peer support roles.

So I think that that barrier will be broken here, ah and I did notice that even on the first week the clinicians wanted to set up their computers, whereas the peer support staff wanted to meet the consumers, and that was like, you kind of look and see which was the priority for people, ah and that clinicians had to really be pulled away from—you don’t need to set up your desk. You know, we want to talk about the consumers. [INTSTA004-CLIN]

Some explicitly foreshadowed difficulties if accepted practices or paradigms were questioned by PSWs, including where those differences reflected disciplinary differences.

Um I guess my big concerns were when it does come time to question things, about having to challenge a little bit, how that’s going to be received. Um so that that will be interesting. [INTSTA050-PEER]

Staff identified that they were unsure about whether the care needs of PSWs would place an additional burden on non-peer staff or risk safe and reliable care (5/15).

The only [ … ] factor that I’d be a bit wary about is uh I suppose the reviewing the risks as well, like after hours. Um how we support our peer support workers if somebody does become acutely unwell, how we support them in that process. [INTSTA045-CLIN]

PSWs identified only one specific need for upskilling, medications management (3/15).

## Discussion

### Discourses of Recovery and Recovery-Oriented Practice

Staff expectations were broadly consistent with the model of service, in terms of their conceptions of recovery and recovery-oriented rehabilitation, although a discussion of medical elements of recovery was relatively uncommon (2). Staff discourses also aligned with the literature on recovery (8, 10, 31, 32). Staff emphasized process-based and individualistic elements of recovery. The ideas that recovery is a personal journey and the importance of fostering empowerment and consumer centered care emerged strongly. References to recovery as a process of individual meaning-making and clinical recovery were uncommon, and the concept of “service-defined recovery” did not emerge (32). As emphasized elsewhere in the recovery literature (33, 34), staff considered that opportunities to facilitate positive risk taking were relevant to fostering empowerment and self-esteem for consumers to assist them in their recovery journey.

Staff expectations aligned with the literature in that they emphasized the importance of focusing on building partnerships and the (consumer) self in recovery-oriented practice (11, 12). Staff also displayed an awareness of challenges that can emerge (3, 4). The prominence of learning and the conceptualization of the CCU as a training ground and simulacrum (or model reality) appear to be somewhat unique with respect to existing literature (11, 12).

The overall understanding and expectations of peer and clinical staff at commencement at a CCU are broadly consistent with those of the consumers entering these services (15, 29). Both consumers and staff focus on recovery as a process, and expected the service to be transitional in nature and to increase consumer independence through skills development. Difference was noted in that, while consumer narratives placed emphasis on the opportunity for personal “transformation,” staff emphasized the role of the CCU as a training ground. This may reflect differences in perspectives between the personal nature of the desired change for consumers in contrast to staff conceptions of their role in facilitating such a change. It is a question for future research whether discourse alignment is maintained over time and contributes positively to achieving therapeutic alliance or greater engagement and outcomes for consumers.

At a structural level, PSW roles were viewed as providing an opportunity for a positive reframing of mental illness, as a vocational strength rather than weakness. This consequence of PSW roles in facilitating positive reframings of mental illness highlights additional benefits to these roles in supporting recovery-oriented practice.

### Identification of Implementation “Success” Factors

Staff identified several known success and risk factors for the implementation of PSW roles within mental health services. The overall coherence in views on recovery and recovery-oriented practice between peer and clinical staff indicates a key support factor for the implementation of PSW roles under the integrated staffing model (22, 35, 36). Staff expressed a high degree of goodwill toward the integrated staffing model, were positively disposed to PSW roles and hoped this model would be successful (14, 17, 20, 21).

### Identification of Implementation “Risk” Factors

Questions of power and equality infused many aspects of staff discourses about how the CCU would evolve. Staff foreshadowed potential challenges to the successful implementation of an integrated model, including the possibility of boundary issues and role conflict (18, 19) as well as the challenge of maintaining role delineation between PSW and clinical roles overtime. While these challenges have been highlighted as implementation “risk factors,” staff also highlighted benefits that could be derived from breaking down barriers and challenging perspectives. This raises questions for future research as to whether awareness of both the benefits and risks of balancing multiple perspectives can serve to prevent conflicts, as well as power imbalances, from developing.

Some staff expressed concerns regarding the vulnerabilities and care needs of PSWs (14, 16, 17). Concerns were raised over job insecurity and poor team dynamics as risks that could undermine goodwill toward either colleagues or residents. The extent to which the temporary nature of the PSW contracts, in comparison to the permanent employment of the clinical staff, impacts the team dynamic will need to be considered in the follow-up interviews. Finally, a few staff identified a lack of role clarity and distinctiveness as possible risks (14, 19–22).

One notable omission in staff discourses was the issue of professionalizing and credentialing the PSW workforce. While professionalizing (and credentialing) PSW roles is a contested issue in the literature, this matter appeared to be a non-issue among those interviewed (14, 17–21). Whether this finding reflects increasing acceptance of the value of PSW by mental health workers or the enthusiasm associated with the commencement of a new service will be explored in planned future research.

### Implications for Practice

Optimism about the integrated staffing model for residential rehabilitation supports the acceptability of this configuration to both clinical and PSWs. It suggests that resistance from clinical staff to the introduction of PSW roles has diminished over time (22) and that PSW roles are increasingly valued. This bodes well for future efforts to increase the level of peer involvement in CCUs and similar services (5, 6).

The extent to which these novel configurations can improve service experiences and outcomes for consumers needs further evaluation. Job security concerns for PSWs remain a challenge, particularly where there is an ongoing debate about the value of PSWs in the empirical literature (13, 37, 38). PSWs at these CCUs were initially employed on a temporary basis as the model was novel and required evaluation, however the positions have since been made permanent. Improved understanding of PSW roles within clinically operated services will enhance employer and service planning confidence in the future.

### Strengths and Limitations

The key strength of this study is that it provides insight into what motivates staff to find work in such services at the beginning of their engagement, and the opportunities and challenges that they foreshadow. However, the overall level of optimism expressed by participants may be due to respondent bias, particularly as staff were new employees and likely desirous to create a good first impression. Alternatively, this positivity may indicate the optimism and excitement about the model of service that compelled their (successful) application for their roles. Hope for a positive experience was tempered by explicit recognition of possible difficulties that lie ahead, indicating critical and honest consideration of the potential realities of the roles.

The CCU was a new service trialing an integrated staffing model that was being added to the existing service landscape. Consequently, inertia or resistance to change among staff at commencement would be unlikely. However, because of this, results may not be generalizable to established services. Future evaluations should examine whether this optimism, tempered by awareness and reflexivity, predicts retention and job satisfaction or, alternatively, whether initial hope and optimism is a risk factor for subsequent disillusionment.

The sampling approach was non-random and approximately one-third of staff working at the services were interviewed, potentially restricting generalizability of findings. However, feedback sessions that included additional staff to those interviewed did not highlight differences or disagreements with the material that had been collected.

## Conclusion

Staff at the CCUs conveyed goodwill and optimism about the integrated staffing model on commencement. This enthusiasm was tempered by realism regarding the potential challenges of recovery-oriented rehabilitation and of integrating peer roles with clinical care. This study supports the acceptability of the integrated staffing model for residential rehabilitation to staff commencing at a CCU, and that PSW roles are valued by both clinicians and people working in lived experience roles. Planned future research to elicit staff experiences, 12–18 months after commencement, will add further insight into the implementation of this model of care, whether, and if so how, views on recovery-oriented rehabilitation change, whether staff priorities and outlooks change, what issues manifest over time, and whether, and if so how, staff attitudes relate to consumer experiences and outcomes.

## Data Availability

The datasets for this study will not be made publicly available because datasets are bound by the confidentiality principles outlined in the ethics approval. Any request for access to the full dataset associated with this project would require further review and approval from the relevant ethics committee.

## Ethics Statement

Ethical clearance was granted by the Metro South Human Research Ethics Committee (HREC/14/QPAH/62).

## Author Contributions

CM contributed to the study design and the training of the interviewer (EN), provided substantial contribution to the analysis, and drafted the initial manuscript. SP contributed to the study design, coordination of the research team, and recruitment at study sites. SP also provided minor contribution to the analysis and reviewed iterative manuscript drafts. EN contributed to the completion of interviews, provided minor contribution to the analysis, and reviewed the initial manuscript draft. FD contributed to the study design, provided minor contribution to analysis, and reviewed iterative manuscript drafts.

## Funding

CM receives salary support from the National Health and Medical Research Council (NHMRC) Centre for Research Excellence in Mental Health Systems Improvement (GNT1041131). SP received a new investigator grant from the Royal Australian and New Zealand College of Psychiatrists.

## Conflicts of Interest Statement

The authors declare that the research was conducted in the absence of any commercial or financial relationships that could be construed as a potential conflict of interest.
